# Piezo 1 and Piezo 2 in the Chemosensory Organs of Zebrafish (*Danio rerio*)

**DOI:** 10.3390/ijms25137404

**Published:** 2024-07-05

**Authors:** Marialuisa Aragona, Kamel Mhalhel, Marzio Cometa, Gianluca Antonio Franco, Giuseppe Montalbano, Maria Cristina Guerrera, Maria Levanti, Rosaria Laurà, Francesco Abbate, José A. Vega, Antonino Germanà

**Affiliations:** 1 Zebrafish Neuromorphology Lab, Department of Veterinary Sciences, University of Messina, 98168 Messina, Italy; kamel.mhalhel@unime.it (K.M.); marzio.cometa@unime.it (M.C.); gmontalbano@unime.it (G.M.); mguerrera@unime.it (M.C.G.); mblevanti@unime.it (M.L.); laurar@unime.it (R.L.); abbatef@unime.it (F.A.); antonino.germana@unime.it (A.G.); 2 Department of Veterinary Sciences, University of Messina, Polo Universitario dell’Annunziata, 98168 Messina, Italy; gianluca.franco@studenti.unime.it; 3 Departamento de Morfología y Biología Celular, Grupo SINPOS, Universidad de Oviedo, 33006 Oviedo, Spain; javega@uniovi.es; 4 Facultad de Ciencias de la Salud, Universidad Autónoma de Chile, Santiago 7500912, Chile

**Keywords:** Piezo 1, Piezo 2, zebrafish, sensory organ, taste buds, isolated chemosensory cells, olfactory epithelium, translational medicine

## Abstract

The ion channels Piezo 1 and Piezo 2 have been identified as membrane mechano-proteins. Studying mechanosensitive channels in chemosensory organs could help in understanding the mechanisms by which these channels operate, offering new therapeutic targets for various disorders. This study investigates the expression patterns of Piezo proteins in zebrafish chemosensory organs. For the first time, Piezo protein expression in adult zebrafish chemosensory organs is reported. In the olfactory epithelium, Piezo 1 immunolabels kappe neurons, microvillous cells, and crypt neurons, while Calretinin is expressed in ciliated sensory cells. The lack of overlap between Piezo 1 and Calretinin confirms Piezo 1’s specificity for kappe neurons, microvillous cells, and crypt neurons. Piezo 2 shows intense immunoreactivity in kappe neurons, one-ciliated sensory cells, and multi-ciliated sensory cells, with overlapping Calretinin expression, indicating its olfactory neuron nature. In taste buds, Piezo 1 immunolabels Merkel-like cells at the bases of cutaneous and pharyngeal taste buds and the light and dark cells of cutaneous and oral taste buds. It also marks the dark cells of pharyngeal taste buds and support cells in oral taste buds. Piezo 2 is found in the light and dark cells of cutaneous and oral taste buds and isolated chemosensory cells. These findings provide new insights into the distribution of Piezo channels in zebrafish chemosensory organs, enhancing our understanding of their sensory processing and potential therapeutic applications.

## 1. Introduction

The sensory systems of fish show specialized sensory organs that include differentiated cells capable of detecting light and mechanical and chemical environment stimuli, with the ability to transform them into electrical signals. Zebrafish (*D. rerio*) have developed sensory mechanisms to detect and process signals essential for survival, feeding, and reproduction. Among these are the chemoreception tasks carried out by taste buds, olfactory organs, and isolated chemosensory cells (ICCC). The distribution and structures of taste buds and the olfactory epithelium [1,2] are well-known in developed and adult zebrafish, which have become attractive and widely employed models in chemoreception studies [3,4,5,6,7,8,9,10]. The zebrafish olfactory organ, located in the dorsal region of its snout, is made from an olfactory epithelium arranged in lamellae that converge in a central raphe that connects the olfactory organ with the aquatic environment. The olfactory epithelium is a pseudostratified columnar tissue mostly made up of supporting and basal cells (no sensory cells) and bipolar neurons known as olfactory sensory neurons (OSNs). Several types of OSNs have been identified in the olfactory epithelium of zebrafish, namely, ciliated (which go through different stages of development depending on the number of cilia) [11], microvillous, crypt, and kappe sensory neurons. Taste buds are chemosensory organs that recognize and evaluate environmental stimuli on the skin, head, lips, and oral cavity [12,13]. Mature taste buds are intraepithelial sensory organs that are pear-shaped and situated on a tiny dermal papilla. In taste buds, two main populations of sensory cells have been identified, and they are known as dark and light cells. In contrast to the latter, which has a single, large microvillus at the apex, the former has an apex with short microvilli. Merkel-like basal cells have been described as being between sensory cells and the basal lamina. Moreover, the skin of zebrafish lips is abundantly covered in ICCCs, which have a spindle-shaped morphology and resemble taste-bud sensory cells. Although the morphology of an ICCC is similar to that of a gustatory receptor cell, these epithelial sensory cells have undergone differentiation and are not classified into organs. The ICCCs we have described are elongated, with extended apical portions reaching the epithelium’s surface and a deep pole directly above the basal layer of the epidermis. The nuclei are positioned basally within them [9,10]. For fish, an aquatic environment is the vehicle for a wide range of stimuli, including smell, oxygen and chemical concentrations, temperature, and physical movement. Survival depends on the ability of an organism to respond and adapt to its surrounding environment; this is possible thanks to the sensory organs. Sensory modes also depend on a combination of ion channels and sensory cells [14,15]. Both intrinsic and extrinsic factors (hormones, chemicals, peptides, membrane potential change, mechanical strength, and temperature changes) are detected by ion channels inducing a signal path that reaches the central nervous system, and from here they are transduced to the visual, olfactory, auditory, gustatory, and somatosensory systems [14] In eukaryotic cells, several ion channels for mechanical forces perception are known to be gated by chemicals, temperatures, and osmolarity [16]. Dysfunction of these ion channels is associated with human disease [17], which highlights the fundamental importance of mechanosensitive channel studies for understanding the mechanotransduction process and finding new therapies and strategies for mechanosensitive disorders. In their study in 2010, Coste et al. [18] identified, for the first time, a new family of mechanically activated channels (Piezo 1 and Piezo 2), which have been proposed as the mechanosensitive ion channels in mammals [19]. Piezo ion channels (Piezo 1 and Piezo 2) are evolutionarily preserved proteins and are fundamental for performing physiological functions in developing and maintaining physiological characteristics and cell volume regulation, migration, proliferation, and elongation [20,21,22]. Piezo proteins have recently been identified as mechanical ion channels that are stretch-activated, localized in the cellular membrane [18,22], and permeable to Ca^2+^ [23], and their roles in sensory and non-sensory epithelia have been observed [18,19]. Piezo proteins could also be involved in the regulation of mechanosensitive flows in sensory neurons caused by inflammatory conditions [24,25,26,27]. Mechanical strength can be transmitted directly to these channels through lateral tension in cellular membranes [28]. Strain involves changes in the thickness of a double layer, resulting in hydrophobic adaptation and subsequent protein configuration-adaptive changes that could open pores [29]. Changes in a membrane’s status or its composition affect the Piezo 1 feature [30]. Piezo orthologues have been identified in numerous eukaryotes. Two-channel isoforms, i.e., Piezo 1 and Piezo 2, have been identified in most vertebrates [18,31], including zebrafish [18,31,32,33,34,35]. In addition, the biophysical properties of piezo proteins in aquatic vertebrates such as *zebrafish* are similar to those of mammalian-dependent voltage channels [24]. Their roles in maintaining homeostasis and cell turnover in zebrafish epithelia have been proven in recent studies [36,37]. Other recent studies [38,39] have suggested the critical roles of Piezo 1 in axonal development and regeneration, as well as the sensory tissue’s fundamental reliance on Piezo 2 [19]. Although studies on piezo proteins in zebrafish have been conducted [18,24,31,32,33,34,35,36,37], little is known about their localization and potential roles in the sensory organs of adult zebrafish. Thus, this study aimed to identify, for the first time, the piezo proteins, Piezo 1 and Piezo 2, in the chemosensory organs of adult zebrafish, which are popular experimental models for translational studies.

## 2. Results

### 2.1. Anti-Piezo 1, Piezo 2, and Calretinin Specificity in Zebrafish

#### 2.1.1. Alignment of Antibody Immunogen Sequences with the Respective Zebrafish Proteins

The anti-Piezo 1, Piezo 2, and Calretinin antibodies are raised against peptides synthesized from their respective human proteins. The alignment of the anti-Piezo 1, anti-Piezo 2, and anti-Calretinin immunogen sequences from human proteins and their respective sequences from zebrafish show that 75.71%, 83.02%, and 72.50%, respectively, of the three peptides’ amino acids match exactly (identity) (Table 1), and this was defined either by their chemical properties or it was based on a point-accepted mutation matrix. The high identity between the antibody immunogens and their respective zebrafish sequences allowed us to hypothesize that the used commercial antibody could be effective on zebrafish.

#### 2.1.2. Western Blot Analyses

**Figure 1:**
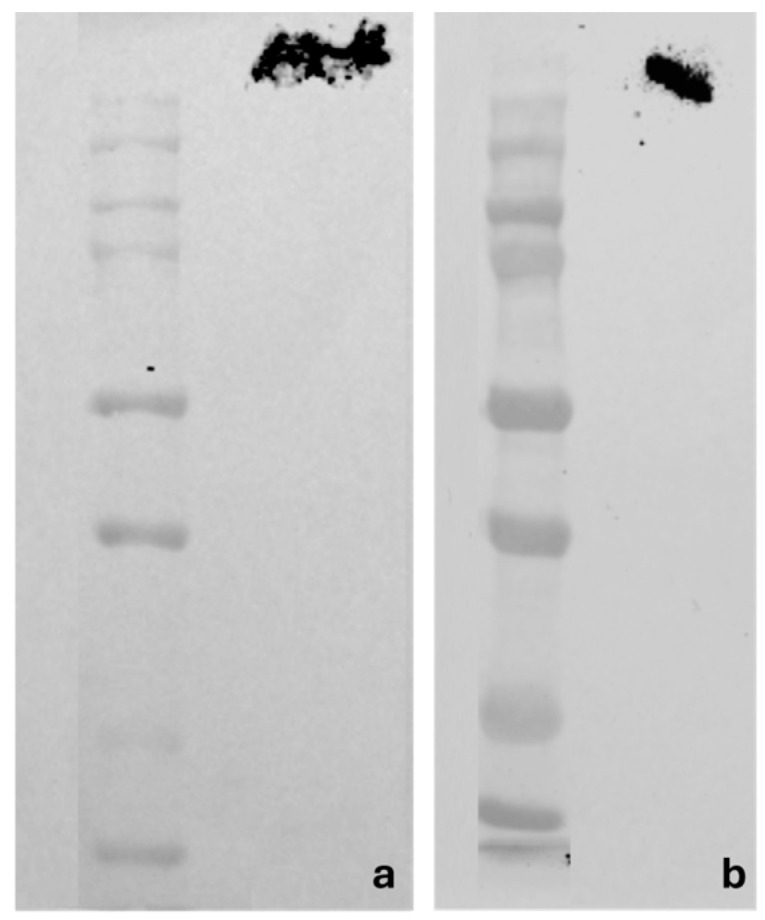
Western blot analyses showing bands corresponding to the molecular weights of the zebrafish Piezo 1 (**a**) and Piezo 2 (**b**) proteins.

The specificity of anti-Piezo 1 and anti-Piezo 2 in zebrafish chemosensory organs was studied using Western blot analyses. The blots of the zebrafish proteins incubated with the anti-Piezo 1 (Cat. # PA5-106296) and anti-Piezo 2 (Cat. # PA5-72975) revealed bands of∼290 and ∼300 KDa, respectively, corresponding to the molecular weights of the zebrafish Piezo 1 and Piezo 2 proteins (Figure 1).

### 2.2. Piezo 1, Piezo 2, and Calretinin Expression Patterns in Zebrafish Chemosensory Organs

**Figure 2:**
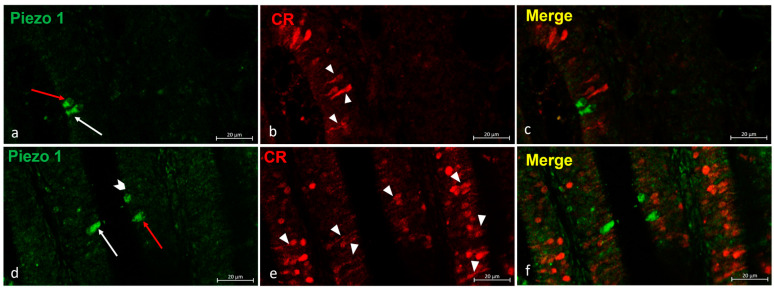
(**a–f**) Zebrafish olfactory lamellae, dorsal view. We conducted an immunohistochemical detection (using the immunofluorescences method) of Piezo 1 in a colocalization view with Calretinin as a specific marker for the neuronal subpopulation cells. (**a**,**d**) The kappe cells (red arrows) and microvillous sensory cells (white arrows) were immunopositive to Piezo 1. (**d**) Piezo 1 was localized in crypt neurons (gallon arrow). (**b**,**e**) The ciliated sensory cells were immunolabeled to Calretinin (arrowhead). (**c**,**f**) A colocalization view that shows no overlap in the labelling. Magnification, 40×; scale bar, 20 µm.

**Figure 3:**
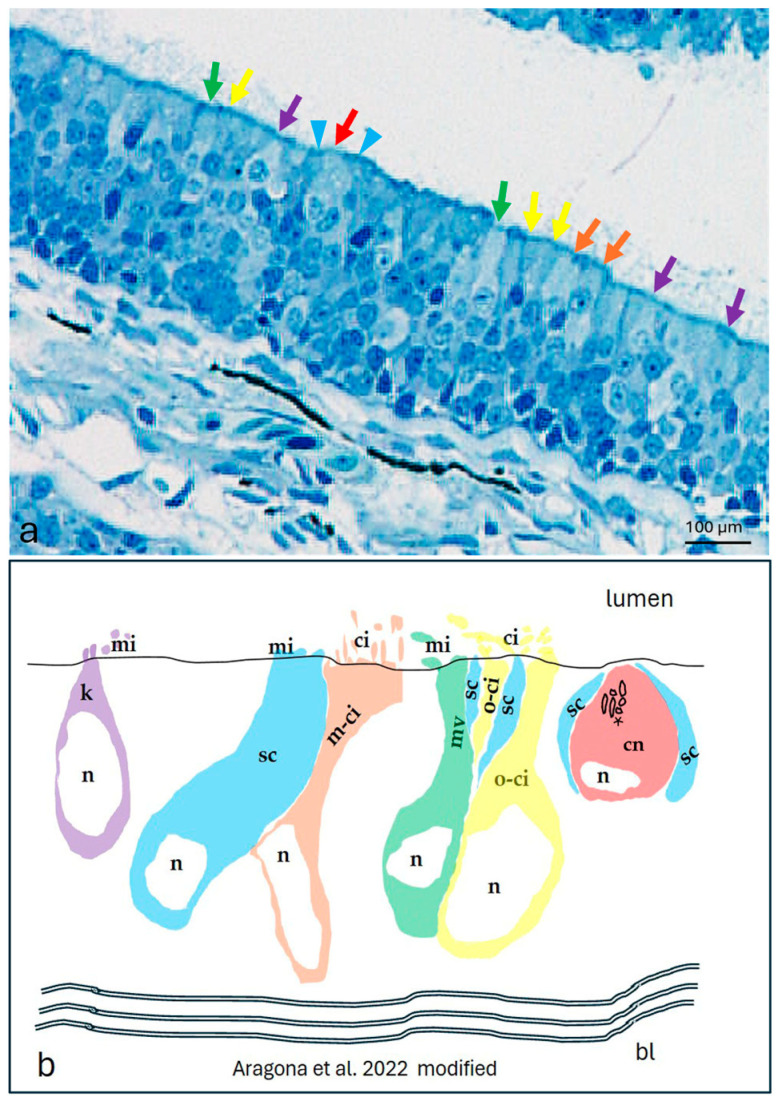
The olfactory epithelium of an adult zebrafish. (**a**) Photomicrographs of the semithin section showing the kappe cells (purple arrows), one-ciliated ON (yellow arrows), multi-ciliated ON (orange arrows), microvillous ON (green arrows), and crypt neurons (red arrow) surrounded by special supporting cells (blue arrowheads). Toluidine blue; magnification, 20×. (**b**) A zebrafish epithelium olfactory neuron’s graphic representation modified using the transmission electron microscopy micrograph from our previous study [10]. Abbreviations: k, kappe cells; sc, supporting cells; m-ci, multi-ciliated ON; n, nucleus; o-ci, one-ciliated ON, ci, cilium; mv, microvillous ON; mi, microvillus; cn, crypt neurons, with several cilia within the crypt (asterisk), special supporting cells (sc), and basal lamina (bl).

An immunohistochemical analysis was carried out on serial sections of adult zebrafish chemosensory organs using the peroxidase method, as well as single and double immunofluorescence. In order to identify the positive cells, we used a morpho-topographical approach based on the observation of the cellular histological features [9,10,41]. Moreover, a colocalization view using Calretinin as a specific marker for neuronal subpopulations in zebrafish sensory epithelia [42] was employed. In the olfactory epithelium of an adult zebrafish, the results showed different neuronal subpopulations with intense Piezo 1 immunoreactivity. Based on the anatomical features (Figure 2d), these cells were identified as kappe neurons, microvillous cells (Figure 2a,d), and crypt neurons. An immunohistochemistry detection of Calretinin in the olfactory epithelium showed an intense immunoreaction in a subpopulation of elongated sensory cells (Figure 2b,e). The lack of overlap between the Piezo 1 and Calretinin labeling (Figure 2 c,f) confirmed the Piezo 1 specificity for kappe neurons, microvillous cells, and crypt neurons. The piezo protein immunolabelled cells were identified based on their anatomical features (Figure 3a,b).

**Figure 4:**
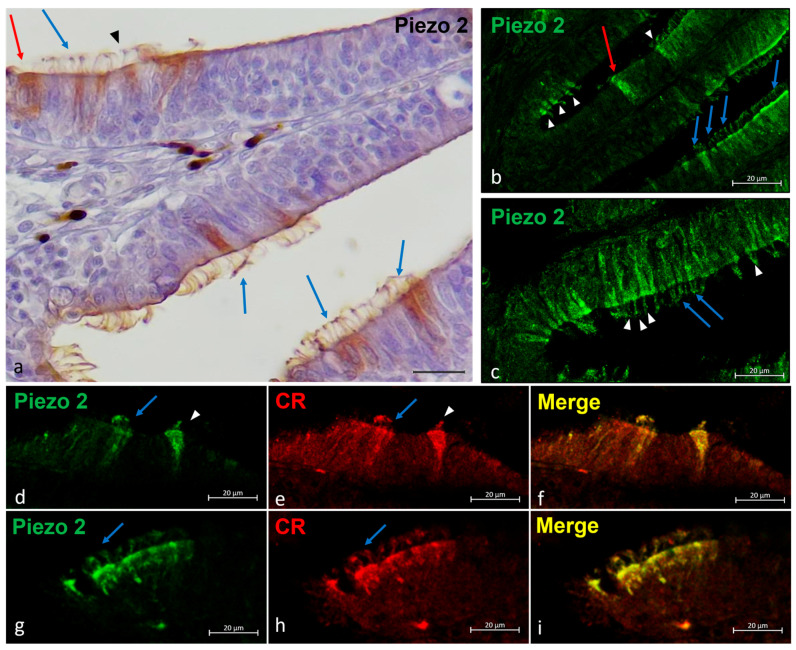
Piezo 2 immunolocalizations in zebrafish olfactory lamellae, dorsal view. (**a**) The immunohistochemical detection (using a peroxidase method, Haematoxylin-stained) of Piezo 2 showing kappe neurons (red arrow), one-ciliated sensory cells (arrowhead), and multi-ciliated sensory cells (blue arrows). (**b**,**c**) The Piezo 2 immunolocalization showing kappe neurons (red arrow), one-ciliated sensory cells (arrowhead), and multi-ciliated sensory cells (blue arrows). (**d**,**e**,**g**,**h**) The immunohistochemical detection of Piezo 2 and Calretinin showing one-ciliated (arrowhead) and multi-ciliated (arrows) sensory cells. (**f**,**i**) A Piezo 2–Calretinin colocalization view. Magnification, 40×; scale bar, 20 µm.

An intense immunoreaction to Piezo 2 was found in some neuronal subpopulations of the olfactory epithelium. Based on a morpho-topographical approach, those cellular subpopulations were identified as kappe neurons, one-ciliated sensory cells, and multi-ciliated sensory cells (Figure 4a–c). Piezo 2 was used in a double experiment with Calretinin as a specific marker for the ciliated sensory cells, and it showed a colocalization in the one-ciliated sensory cells (Figure 4d–i).

**Figure 5:**
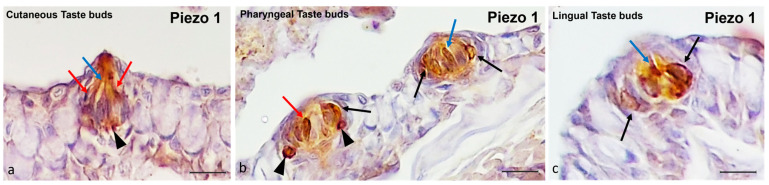
Zebrafish taste buds, transversal view. The immunohistochemical detection (using a peroxidase method) of Piezo 1. (**a**) In the cutaneous taste buds, the Merkel-like cells (arrowheads), light cells (blue arrows), and dark cells (red arrows) showed immunopositivity to Piezo 1. (**b**) In the pharyngeal taste buds, the Merkel-like cells (arrowheads), light cells (blue arrows), dark cells (red arrows), and supporting cells (black arrows) showed immunoreactivity to Piezo 1. (**c**) In the lingual taste buds, the light cells (blue arrow) and supporting cells (black arrows) were Piezo 1-immunolabelled. Magnification, 40×; scale bar, 20 µm.

The immunohistochemical detection of Piezo 1 in zebrafish taste buds showed intense immunoreactivity (Figure 5). Using a histological and morpho-topographical approach, Piezo 1 was found in the Merkel-like cells at the bases of the cutaneous and pharyngeal taste buds (Figure 5a,b), as well as in the light cells of the cutaneous and oral taste buds (Figure 5a–c). Moreover, Piezo 1 marked the dark cells in the cutaneous and pharyngeal taste buds (Figure 5a,b) and supported the cells in the oral taste buds (Figure 5b,c).

**Figure 6:**
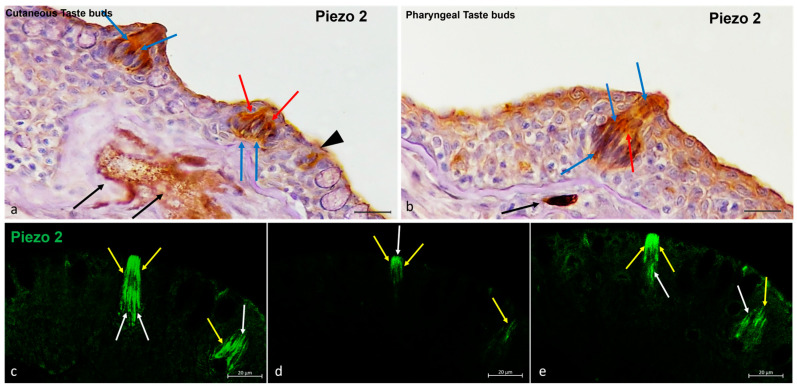
Zebrafish taste buds, transversal view. (**a**,**b**) The immunohistochemical detection (using a peroxidase method, Haematoxylin-stained) of Piezo 2. The light cells (blue arrows), dark cells (red arrows), and nerve cells (black arrows) were Piezo 2-immunopositive. (**a**) Isolated chemosensory cells (black arrowhead) were immunoreactive to Piezo 2. (**c**–**e**) The multiple focal planes of the Piezo 2 immunohistochemical detections (using a fluorescence method). The light cells (white arrows) and dark cells (yellow arrows) were immunoreactive to Piezo 2. Magnification, 40×; scale bar, 20 µm.

Piezo 2 was found in the cutaneous and oral taste buds (Figure 6). The immunostained cellular subpopulations were identified as light cells and dark cells (Figure 6a,b). Even the nerve innervating the taste buds exhibited immunopositivity for Piezo 2 (Figure 6a,b). In addition, the isolated chemosensory cells were Piezo 2-immunolabeled (Figure 6a). Finally, the fluorescence multiple focal planes showed Piezo 2 immunoreactivity in the light and dark cells from the basis to the cellular apex (Figure 6c,d).

**Figure 7:**
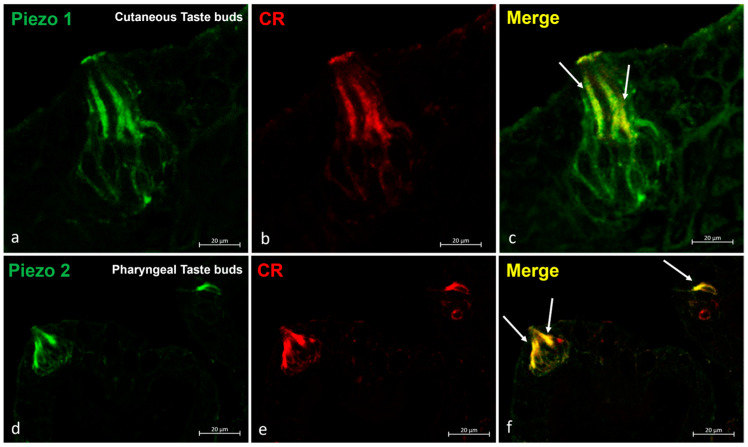
Zebrafish taste buds, transversal view. The immunohistochemical detection (using fluorescence methods) of Piezo 1 and Piezo 2. (**a**–**c**) The double experiments with Piezo 1 and Calretinin showed an overlapping stain in the sensory cells (arrows) of the cutaneous taste buds. (**d**–**f**) The double experiments for Piezo 2 and Calretinin showed an overlapping stain in the sensory cells (arrows) of the pharyngeal taste buds. Magnification, 40×; scale bar, 20 µm.

In order to ascertain the sensorial nature of the Piezo 1 and Piezo 2 immunoreactive cells, piezo proteins were used in a double experiment with Calretinin as specific markers for the taste buds and sensory cells. In this way, overlapping immunostaining was observed (Figure 7).

### 2.3. Statistical Analysis

**Figure 8:**
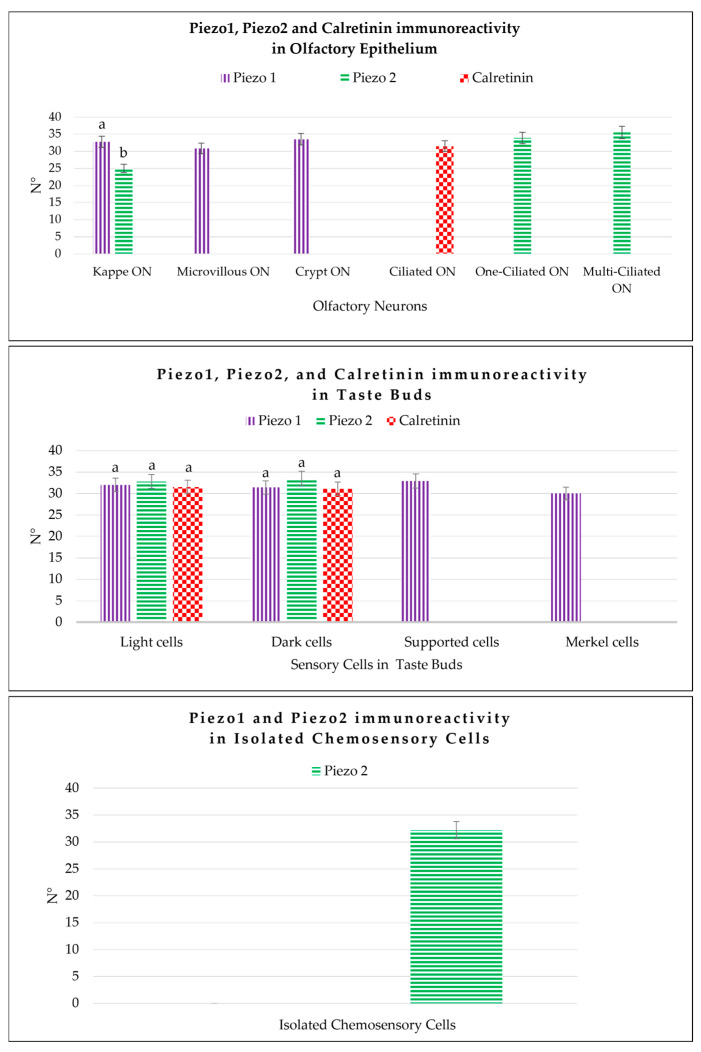
Graphical representation of the immunoreactive cell counts: the kappe olfactory neurons (Kappe ON), microvillous olfactory neurons (Microvillous ON), crypt olfactory neurons (Crypt ON), one-ciliated olfactory neurons (One-ciliated ON), and multi-ciliated olfactory neurons (Multi-ciliated ON) in the olfactory epithelium labeled by Piezo 1, Piezo 2, and Calretinin; the light cells, dark cells, Merkel-like cells, and supporting cells of the taste buds immunolabeled by Piezo 1, Piezo 2, and Calretinin; and the isolated chemosensory cells marked by Piezo 2. The statistical analysis showed different expression patterns for the investigated proteins in the different cellular subpopulations. N°, mean of the cells immunopositive to Piezo 1, Piezo 2, and Calretinin. The lowercase letters indicate the statistical significance between the different cell subpopulations, with *p* < 0.05.

According to the results of the quantitative analysis, Piezo 1 and Piezo 2 were immunolocalized in the kappe neurons, and the Piezo 1-immunolabeled cells were greater than those for Piezo 2. Piezo 1 was also detected in microvillous cells and crypt neurons, but Piezo 2 was not. Piezo 2 immunolabeling was noted in the sensory cells and in the one- and multi-ciliated in the olfactory epithelium. In the zebrafish taste buds, the Piezo 1 and Piezo 2 labeling showed a comparable number of light and dark cells, while the supporting cells and the Merkel-like cells were only Piezo 1-positive. Other than the taste bud cells, Piezo 2 immunolabeled the isolated chemosensory cells. Finally, Calretinin, when used as a specific marker for the sensory cells in the olfactory epithelium and taste buds, showed immunoreactivity in these organs. A comparison of the piezo proteins, Piezo 1, Piezo 2, and Calretinin immunoreactivity in the different cell subpopulations of zebrafish olfactory epithelia, taste buds, and isolated chemosensory cells is shown in Figure 8 and Table 2 and Table 3.

## 3. Discussion

Ion channels detect environmental stimuli, creating a signal stream that reaches the central nervous system from where they are transduced to the visual, olfactory, auditory, gustatory, and somatosensory systems [14]. The ability to respond to environmental stimuli is crucial to evolution and survival [14]. Zebrafish have evolved intricate sensory systems to recognize and interpret signals vital to survival, eating, and procreation, including taste buds, an olfactory organ, and isolated chemosensory cells (ICCC) [10]. The zebrafish is an established model for studying human diseases thanks to their ability to regenerate and their continuous growth until adulthood, as well as their competence in maintaining an intrinsic plasticity [43,44]. It represents a suitable model for translational studies of sensory organ dysfunction, in particular, for investigating changes in taste and smell resulting from disease (including viral infections such as COVID-19 [9]) and/or traumatic events, as well as their potential regeneration [9]. During the regenerative events, ion channels appear to play a key role [45,46] among various other factors (growth factors, ions, etc.) [10]. They are implicated in maintaining homeostasis and cell turnover in zebrafish epithelia [36,37]. Although multiple studies on zebrafish piezo proteins [18,21,31,32] have been conducted [24,33,34,35,36,37], their localization and potential roles in the sensory organs of adult zebrafish remain inadequately understood [45]. Therefore, this study aimed to conduct an immunohistochemical investigation to identify Piezo 1 and Piezo 2 proteins in the chemosensory organs, including the taste buds, olfactory sensory epithelia, and ICCCs, of zebrafish. In addition to determining the expression patterns of Piezo 1 and Piezo 2 in the three chemosensory structures, Calretinin immunolabeling, which involves a specific marker of sensory ciliate cells of the chemosensory epithelia of zebrafish, was conducted [9,10,42,47]. To the authors’ best knowledge, there is currently no evidence of a specific marker for identifying the microvillus olfactory neurons and kappe olfactory neurons at different stages of maturity in zebrafish. For the ciliate neurons, our research group [10,42] has introduced Calretinin as a specific marker for the zebrafish olfactory ciliate neurons.

Since the authors’ use of the antibodies’ specificity on zebrafish was not guaranteed by the manufacturer, and since no previous studies have reported on this, the ability of the antibodies to recognize their immunogens on zebrafish tissue was confirmed by both the high identity of the antigen sequences with their respective zebrafish proteins and by the bands corresponding to the zebrafish piezo proteins’ molecular weights. This study reported, for the first time, the localization of the piezo proteins Piezo 1 and Piezo 2 in the chemosensorial epithelia of zebrafish. In the olfactory epithelium, the microvillous, kappe, and crypt olfactory neurons were Piezo 1-immunopositive. Moreover, only ciliated cells were Piezo 2-immunolabelled. In particular, these Piezo 2-immunopositive cells exhibited variations in both cilia length and number, differentiating between the diverse olfactory neurons of different stages of maturity. This observation was confirmed by recent studies conducted on zebrafish, where immature olfactory neurons had few cilia, while the mature ones had numerous cilia [11]. Taken together, these findings highlight that the immature and mature olfactory neurons in the adult zebrafish specimens were Piezo 2-immunoreactive. In addition, the double-stained Piezo 2/Calretinin neurons confirmed the olfactory neuron nature of the Piezo 2-immunoreactive cells. In light of what is known and considering that no olfactory neurons were Piezo 1- or Calretinin-double-labeled, Piezo 1 could be considered as an antibody marker for kappe and microvillous olfactory neurons. However, Piezo 2 could be considered as a marker for ciliate olfactory neurons at different stages of maturity. Piezo 2’s ability to label the ciliate neurons’ cilia could be a promising avenue for elucidating the stages of maturation and regeneration in zebrafish olfactory epithelia. In taste buds, different immunolocalizations were observed for Piezo 1 and Piezo 2. Light and dark cells were Piezo 1- and Piezo 2-immunolabelled, both in the cutaneous and in the lingual and oropharyngeal taste buds. The Merkel-like cells in the skin and pharyngeal taste buds were Piezo 1-immunostained, while the ICCCs were exclusively Piezo 2-immunoreactive. Our findings were reminiscent of those of Moayedi et al. (2018), where they revealed the expression of Piezo 2 in Merkel-like cells or in Merkel-like cell afferents, as well as in the end bulbs of Krause, and they identified a novel class of neuronal fibers innervating the epithelia surrounding the taste buds [48]. A recent study has confirmed the findings of Moayedi et al. and suggested a new role for Piezo 2 in mediating food texture sensation in mammals [48]. The taste bud cells’ Piezo- and Calretinin-double-staining, known sensory cellular markers for zebrafish taste buds [10,42], confirmed the sensory cell nature of the piezo protein-immunoreactive cells. These data are intriguing since, for the first time, they report on Piezo 1 and Piezo 2 in chemosensory cells with chemosensorial functions. Indeed, in mammalian taste buds, different types of ion channels are involved in chemoreception. In fish, ion channels are similarly involved in taste perception and are present in taste buds [15]. Thus, the Piezo 1 and Piezo 2 immunoreactivity in the sensory cells of zebrafish taste buds shown in this study suggest that piezo proteins function similarly to other ion channels [14,15], participating in the signal transduction and/or the synaptic release of chemical transmitters. Indeed, Piezo 1 and Piezo 2 are known to be implicated in the regulation of the Ca^2+^ inflow and, thus, in numerous physiological functions, including signal transduction, neurotransmitter release, and cellular excitability. Piezo proteins have also been shown to regulate neurological development processes, axon regeneration, and neurogenesis in human neural stem cells [49]. It has recently been observed that piezo proteins instruct the targeting of olfactory projection neurons, and the activity of the mechanically activated ion channel is superfluous for this function, suggesting that piezo proteins can also function independently of mechanosensible ion channels thanks their piezoelectric molecular partners. The systematic identification of piezo proteins’ molecular partners can reveal how piezo proteins instruct dendrite targeting and how they work regardless of the activity of their mechanistic channels [50]. Taken together, the results shown and the recent discoveries on the functions and actions of Piezo 1 and Piezo 2 open new scenarios for the roles of these important ionic membrane channels.

## 4. Materials and Methods

### 4.1. Piezo 1, Piezo 2, and Calretinin Specificity in Zebrafish

#### 4.1.1. Blast of the Antibody Immunogen Sequences with the Respective Zebrafish Proteins

The Piezo 1, Piezo 2, and Calretinin antibodies (for details, see Table 1) were raised against peptides synthesized from the respective human proteins. In order to verify those primary antibodies’ specificity to the zebrafish proteins, a protein alignment was performed for the different antibody immunogen sequences from human proteins and their respective sequences from the zebrafish. The online software NCBI blastp (protein-protein BLAST https://www.ncbi.nlm.nih.gov/) was used for the peptide alignment [51].

#### 4.1.2. Western Blot Analyses

Western blot analyses were performed as previously described [52] on the zebrafish head homogenates. The anti-Piezo 1 and anti-Piezo 2 primary antibodies (Table 4) were used.

### 4.2. Sample Treatment

The adult zebrafish specimens were maintained using routine procedures [9,10,41]. All animal handling protocols were carried out in accordance with the principles outlined in the Declaration of Helsinki and approved by the Italian Ministry of Health (A.M. n. 505/2023-PR). Tissue samples from the fresh specimens were fixed in 4% paraformaldehyde in phosphate-buffered saline (PBS) (AAJ19943K2, Thermo Scientific, Waltham, MA, USA) 0.1 m (pH = 7.4) for 12–18 h, dehydrated through a graded ethanol series, and clarified in xylene for paraffin-wax-embedding. The embedded tissue samples were then cut into 7 µm-thick serial sections and collected on gelatin-coated microscope slides. Furthermore, a semi-thin section (0.99 µm) from a previous study [41] was stained with Toluidine Blue (Sigma-Aldrich, Saint Louis, MO, USA cat#T3260), dehydrated, mounted, and examined under a Leica DMRB light microscope equipped with a Leica MC 120 HD camera (Leica Application Suite LAS V4.7 Leica Microsystems GmbH, Wetzlar, Germany).

### 4.3. Immunohistochemistry

#### 4.3.1. Peroxidase Method

To analyze the expression of Piezo 1 and Piezo 2 in the sensory patches of the chemosensory system (taste buds and olfactory lamellae) of the adult zebrafish, serial sections were deparaffinized and rehydrated, washed in working buffer (Tris–HCl buffer 0.05 M, pH 7.5) containing 0.1% bovine serum albumin and 0.2% Triton-X 100, and incubated in 0.3% H_2_O_2_ (PBS) solution for 3 min to prevent the activity of endogenous peroxidase. Then, fetal bovine serum (F7524 Sigma-Aldrich) was added to the rinsed sections for 30 min to avoid non-specific binding. The incubation with Piezo 1 and Piezo 2 rabbit polyclonal antibodies was carried out overnight at 4 °C in a humid chamber. Afterward, the incubated sections were washed in the working buffer and incubated for 1.5 h at room temperature with a secondary antibody-peroxidase conjugate (see Table 4). The immunoreaction was visualized using 3-30-diaminobenzidine as a chromogen (DAB, Sigma-Aldrich, Inc., St. Louis, MO, USA cat. #D5905). Finally, the slides were counterstained with Haematoxylin (Bio-Optica Milano S.p.a Italy cat. # 05-M06012), dehydrated, mounted, and examined under a Leica DMRB light microscope equipped with a Leica MC 120 HD camera (Leica Application Suite LAS V4.7 Leica Microsystems GmbH, Wetzlar, Germania).

**Table 4:** Antibodies used in the study.

Primary Antibodies	Supplier	Catalogue Number	Source	Dilution	Antibody ID
Piezo 1	Invitrogen-Thermo Fisher Scientific, Waltham, MA USA	PA5-106296	rabbit	1:100	AB_2853973
Piezo 2	Invitrogen-Thermo Fisher Scientific, Waltham, MA USA	PA5-72975	rabbit	1:100	AB_2718829
Calretinin	Santa Cruz Biotechnology, Inc., Dallas, Texas, USA	sc-11644	goat	1:100	AB_634545
**Secondary** **Antibodies**	**Supplier**	**Catalogue Number**	**Source**	**Dilution**	**Antibody ID**
anti-Goat IgG (H + L) Alexa Fluor 594	Invitrogen-Thermo Fisher Scientific, Waltham, MA USA	A-11058	donkey	1:300	AB_2534102
anti-Rabbit IgG (H + L) Alexa Fluor 488	Invitrogen-Thermo Fisher Scientific, Waltham, MA USA	A-11008	goat	1:300	AB_143165
anti-rabbit IgG-peroxidase conjugate	Amersham bioscences, Piscataway, NJ, USA	NA934	donkey	1:100	AB_772206

#### 4.3.2. Confocal Immunofluorescence

Some serial sections were treated as described above and then incubated with primary antibodies. The Calretinin goat polyclonal antibody was used in the double-label experiments with the anti-Piezo 1 and anti-Piezo 2 polyclonal antibodies (see Table 4). Incubation was carried out overnight at 4 °C in a humid chamber. After rinsing in working buffer, the sections were incubated for 1 h at room temperature in a dark, humid chamber with anti-Goat IgG (H + L) Alexa Fluor 594 and anti-rabbit IgG (H + L) Alexa Fluor 488 secondary fluorescent antibodies (see Table 4). Finally, the sections were washed, dehydrated, and mounted with Fluoromount™ Aqueous Mounting Medium (Sigma Aldrich, Saint Louis, MO, USA). The sections were analyzed and images were acquired using a Zeiss LSMDUO confocal laser scanning microscope with a META module (Carl Zeiss MicroImaging GmbH, München, Germany) microscope (LSM700 AxioObserver Carl Zeiss MicroImaging GmbH, München, Germany). A Zen 2011 (LSM 700 Zeiss software ZEN 3.7 Carl Zeiss MicroImaging GmbH, München, Germany) built-in “colocalization view” was used to highlight the expression of both antibody signals to produce a “colocalization” signal, the scatter plot, and the fluorescent signal measurements. Each image was rapidly acquired to minimize photodegradation. To provide negative controls, representative sections were incubated with specifically preabsorbed antisera as described above. Under these conditions, no positive immunostaining was observed.

#### 4.3.3. Cell Counting

Cell counts were performed using ImageJ (ImageJ, U. S. National Institutes of Health, Bethesda, MD, USA, https://imagej.nih.gov/ij/, version 1.53). Immunofluorescent microphotographs were scaled to μm and converted to grayscale, and artifacts were removed by adjusting the threshold. An area tool was used to select the region of interest. Cell numbers were expressed as counts per organ.

### 4.4. Statistical Analysis

ImageJ software was used to evaluate the randomly collected microscope fields [53]. One-way ANOVA was used to examine the statistical significance of the quantities of the neuronal subpopulations in the zebrafish chemosensory epithelia (the olfactory epithelium and taste buds) detected by Piezo 1, Piezo 2, and Calretinin. SigmaPlot version 14.0 (Systat Software, San Jose, CA, USA) was used to conduct the statistical analysis. An unpaired Z test was also performed. The information was given as mean values with standard deviations (Δσ). Values of *p* below 0.05 were considered statistically significant (*p* < 0.05).

## 5. Conclusions

This study reported, for the first time, the localization of Piezo 1 and Piezo 2 in the chemosensory organs of the adult zebrafish. Although piezo proteins have always been considered mechanosensitive proteins, several of their functions have been recently discovered that are not limited to mechanotransduction, namely, their roles in olfactory projections. Unfortunately, nothing is yet known about their roles in taste organs. The current results aim to lay the basis for discovering new roles and functions of piezo proteins in chemotransduction. Future studies are needed using zebrafish piezo-transgenic models for translational medicine research on sensorial disorders other than those in the channelopathies resulting from disease, infection, and/or injury to reveal the potential roles of piezo proteins in sensory and developmental processes.

## Figures and Tables

**Table 1:** Comparison of Piezo 1, Piezo 2, and Calretinin identity between humans and zebrafish.

Antibodies		Taxa’s Protein		Report of the Multiple Alignment					
Product	Immunogen	Taxa	Query ID	Max Score	Total Score	Query Cover	E-Value	Per. Ident.	Acc. Len.
Piezo 1 Cat. # PA5-106296	Human Piezo 1 (accession Q92508), corresponding to amino acid residues I642-L692	*Danio rerio*	XP_696355.4	120	120	2%	1 × 10^−37^	75.71%	70
				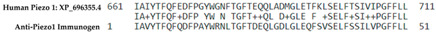					
Piezo 2 Cat. # PA5-72975	Human Piezo 2 protein (between residues 1450 and 1500) [UniProt Q9H5I5]	*Danio rerio*	XP_021323952	93.6	93.6	1%	4 × 10^−28^	83.02	53
				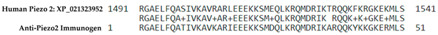					
Calretinin (N-18) Cat. # sc-11644	The specificity of the antibody has been previously demonstrated [10,40].								

**Table 2:** Mean data ± standard deviation (∆σ) of the immunoreactive cells counts for the following: kappe neurons, microvillous cells, one-ciliated cells, and multi-ciliated cells of the olfactory epithelium detected by Piezo 1, Piezo 2, and Calretinin. The statistical analysis showed different expression patterns for the investigated proteins in the different cellular subpopulations. Mean values ± standard deviation, Δσ; -, no immunoreactivity detected.

	Mean ± ∆σ of Kappe ONs	Mean ± ∆σ of Ciliated ONs	Mean ± ∆σ of One-Ciliated ONs	Mean ± ∆σ of Multi-Ciliated ONs	Mean ± ∆σ of Microvillous ONs	Mean ± ∆σ of Crypt ONs
Piezo 1	32.8 ± 4.6	-	-	-	30.8 ± 3.1	33.5 ± 4.9
Piezo 2	25 ± 3.9	-	33.9 ± 3	35 ± 4.9	-	-
Calretinin	-	31.5 ± 4.5	33.5 ± 2.6	36 ± 8.7	-	-

**Table 3:** Mean data ± standard deviation (∆σ) of the immunoreactive cell counts for the following: light cells, dark cells, Merkel-like cells, and supporting cells of the taste buds detected by Piezo 1, Piezo 2, and Calretinin, as well as the isolated chemosensory cells detected by Piezo 2. The statistical analysis showed different expression patterns for the investigated proteins in the different cellular subpopulations. Mean values ± standard deviation, Δσ; -, no immunoreactivity detected.

	Mean ± ∆σ of Light Cells	Mean ± ∆σ of Dark Cells	Mean ± ∆σ of Supporting Cells	Mean ± ∆σ of Merkel-Like Cells	Mean ± ∆σ of ICCCs
Piezo 1	32 ± 5.9	31.4 ± 4.7	32.9 ± 3.7	30 ± 6.8	-
Piezo 2	28.4 ± 6	33.5 ± 3.8	-	-	32.2 ± 2.7
Calretinin	29.7 ± 3.9	31.1 ± 3.1	-	-	-

## Data Availability

All data presented this study are available from the corresponding author upon responsible request.
